# Differential Studies on the Structure of Lignin–Carbohydrate Complexes (LCC) in Alkali-Extracted Plant Hemicelluloses

**DOI:** 10.3390/polym16101403

**Published:** 2024-05-15

**Authors:** Shuyu Pang, Xin Wang, Jiali Pu, Chen Liang, Shuangquan Yao, Chengrong Qin

**Affiliations:** Guangxi Key Laboratory of Clean Pulp & Papermaking and Pollution Control, School of Light Industrial and Food Engineering, Guangxi University, Nanning 530004, China; 2116301042@st.gxu.edu.cn (S.P.); 2016301031@st.gxu.edu.cn (X.W.); 2216391035@st.gxu.edu.cn (J.P.); yaoshuangquan@gxu.edu.cn (S.Y.); qinchengrong@gxu.edu.cn (C.Q.)

**Keywords:** Gramineae, phenyl glycosidic bonds, alkaline environment, lignin–carbohydrate complex (LCC)

## Abstract

Hemicellulose extracted by alkali treatment is of interest because of the advantages of its intact sugar structure and high degree of polymerization. However, the hemicellulose extracted by alkali treatment contained more lignin fragments and the presence of a lignin–carbohydrate complex (LCC), which affected the isolation and purification of hemicellulose and its comprehensive utilization. Therefore, the evaluation of the LCC structure of different types of lignocellulosic resources is of great significance. In this study, the LCC structures of hardwoods and Gramineae were enriched in alkaline systems. Information on the composition, structural proportions, and connection patterns of LCC samples was discussed. The similarities and differences between the LCC structures of different units of raw materials were comparatively studied. The results indicated that the monosaccharide fractions were higher in the LCC of Gramineae compared to hardwoods. The composition of the lignin fraction was dominated by G and S units. The phenyl glycosidic (PhGlc) bond is the predominant LCC linkage under alkali-stabilized conditions. In addition, Gramineae PhGlc types are more numerous compared to hardwoods. The results of the study provide insights into the differences in the chemical composition and structural features of LCC in different plants and provide important guidance for the optimization of the process of purifying hemicellulose.

## 1. Introduction

Lignocellulosic biomass has gradually become considered a high-quality substitute for fossil resources due to its advantages of being renewable and having a short growth cycle [1]. Therefore, the whole-component separation of lignocellulosic biomass can improve its utilization efficiency [2]. Currently, the alkali treatment method has been widely focused on as a common method for the separation of biomass components [3]. The alkaline extraction of hemicellulose has the advantages of high molecular weight, low-branched hemicellulose with intact sugar structure, a higher degree of polymerization, and a higher yield [4]. During the alkali treatment, both hemicellulose and lignin are leached out in large amounts due to their high solubility, while the cellulose portion is retained. The alkali pretreatment process causes cellulose swelling, which changes the structure of lignocellulose, leading to a decrease in its crystallinity. Moreover, the process of delignification by alkali pretreatment is effective for biomass with low lignin content, but relatively inefficient for hardwood biomass [5]. Solubilized hemicellulose has been interspersed with lignin. Cross-linking between lignin and hemicellulose to form a lignin–carbohydrate complex (LCC) hinders the separation and purification of hemicellulose and affects the high-value utilization of hemicellulose. Therefore, it is beneficial to effectively reduce the recalcitrant structure of biomass and improve the utilization of Agroforestry biomass by analyzing the composition and structure of LCC [6].

**Figure 1:**
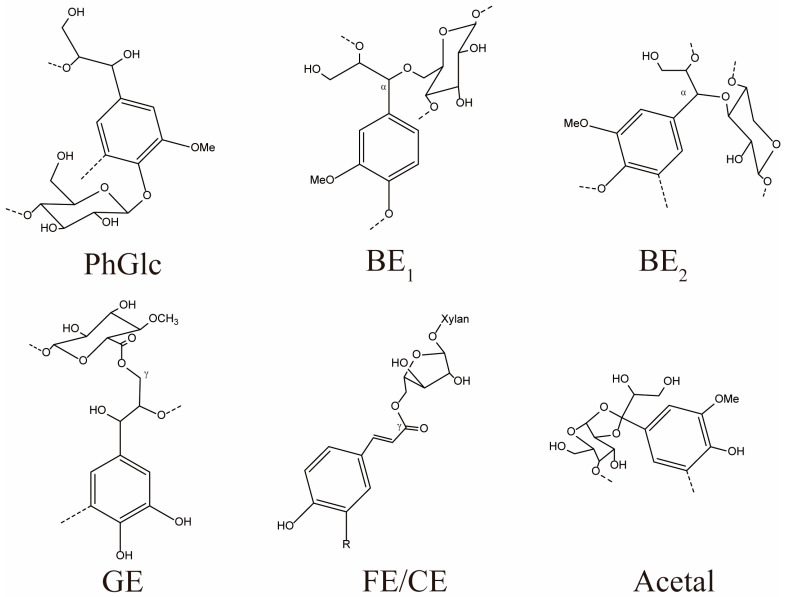
Main types of LC bonding. (PhGlc = phenyl glycosides, BE = benzyl ethers, GE = γ-esters, FE = ferulate esters, CE = conmarate esters).

It is crucial to study lignin–carbohydrate (LC) bonding during the exploration of the LCC structure. LC bonds are usually formed by the connection between lignin and carbohydrates through carbon–oxygen bonds (as shown in Figure 1), such as phenyl glycosidic (PhGlc) bonds, benzyl ether (BE) bonds, γ-ester (GE) bonds, hemiacetal or acetal bonds, and ferulic (FA) and *p*-coumaric (*p*CA) ester bonds [7]. The PhGlc bond is primarily a side-chain hydroxyl group connecting carbohydrates to lignin. The BE bond is primarily a sugar group of carbohydrates connected to a phenolic or hydroxyl group of lignin. The GE bond is primarily an aldehyde group of sugar connected to a hydroxyl group of lignin. The acetal bond is formed by joining the carbonyl group on the phenylpropane structure of lignin to the hydroxyl group of the carbohydrate. Large amounts of ferulic and *p*CA bind to carbohydrates in the cell wall. FA are a major component of LCC in graminoids and other non-woody plants [8]. Cui et al. [9] used isotopic labeling to analyze the morphology and structure of poplar wood enzymatic LCC and discovered that the LC bonds mainly included ester, ether, and acetal bonds. In addition, the structure and connection of LC bonds differed under different treatment environments, thus resulting in different structures of LCC. For instance, in alkaline environments, the saponification reaction of sodium hydroxide usually causes the breakage of ester bonds in LCC linkage bonds. Wang et al. [10] enriched the LCC structure in bamboo under alkaline conditions and found that the linkage bonds of LCC in bamboo mainly included PhGlc bonds. Moreover, Feng et al. [11] extracted LCC from poplar wood by using hydrothermal pretreatment and detected that the LC bonds in the wood were mainly dominated by phenyl glycoside bonds and BE bonds. Related studies have shown that there are also differences in the LCC structures of different kinds of raw materials, except for the effect of treatment conditions on the information of LCC components and connection methods [12]. Zhao et al. [13] demonstrated that for giant eucalyptus enzymatic LCC, the LC bonds mainly included BE bonds, and no PhGlc bonds were present. Furthermore, Balakshin et al. [14] observed the presence of small amounts of PhGlc and GE bonds in pine enzymatic LCC.

Gramineae plants have advantages such as a short growth cycle compared to wood, making their utilization and development indispensable [15]. Related studies have demonstrated the presence of large quantities of hemicellulose and lignin in Gramineae. Therefore, more LCC structures are also present, which affect their full-component utilization. Huang et al. [16] observed that LCC and residual xylan in bamboo pulp can hinder its enzymatic digestibility. In addition, Yang et al. [17] found a small amount of LCC structure in wheat straw by treating it in sulfate slurry after initial treatment, which adversely affected the subsequent enzymatic digestion. The abovementioned study indicated that the presence of LCC hinders the bleaching of pulp and the further utilization of carbohydrate fractions. However, LCC has excellent properties in terms of oxidation resistance that can give it special properties in the preparation of related materials. Xie et al. [18] investigated the structure of LCC in wheat straw and explored the antioxidant capacity of LCC, and their data provide a theoretical basis for the subsequent development and utilization of LCC. Hence, the investigation of the LCC structure in Gramineae is essential to decipher information on their resistance to deconjugation.

In this article, the LCC structure was enriched and analyzed via alkali treatment using Gramineae (bagasse, bamboo, and corn straw) and hardwoods (eucalyptus and acacia) as raw materials to evaluate the LCC component composition, structural ratio, and connection mode in different types of raw materials. The compositional and molecular weight sizes of LCC were analyzed by high-pressure ion chromatography (IC), ultraviolet spectrophotometry (UV), and gel permeation chromatography (GPC). Moreover, nuclear magnetic resonance spectroscopy (NMR) was used for qualitative and semiquantitative analyses of the LCC structure. The results demonstrated the differences in LCC structures between hardwoods and Gramineae and provided a data reference for exploring LCC structure studies of Gramineae under alkaline environments.

## 2. Materials and Methods

### 2.1. Experimental Materials

Gramineae and hardwoods were used as experimental raw materials, including bagasse (Nanning, Guangxi, China), bamboo (Nanning, Guangxi, China), corn straw (Lianyungang, Jiangsu, China), acacia (Nanning, Guangxi, China), and eucalyptus (Nanning, Guangxi, China). The five experimental raw materials were crushed through a grinder (P-15, FRITSCH, Idar-Oberstein, Germany), screened for wood powder below 40 mesh, and air-dried. Xyloglucan endonuclease, XAD-16N macroporous resin, and DMSO-d_6_ (spectral grade) were purchased from Sigma Reagent Company (Burlington, MA, USA). Additionally, anhydrous ethanol, sodium hydroxide, hydrochloric acid (36%), methyl alcohol, citric acid, and sodium citrate were purchased from Aladdin Company (Shanghai, China) (all labeled reagents are of analytical grade).

### 2.2. Extraction of Hemicellulose via the Alkali Method

The process of alkaline hemicellulose extraction was conducted according to the previous research method of our group, which was described below [4]. Five different absolutely dry raw materials (120 g) were weighed. The raw materials were fully immersed in distilled water for 12 h at 25 °C. The solid-liquid ratio was 1:10. In addition, a certain amount of 1 mol·L^−1^ NaOH solution was added according to the 5% alkali concentration. After soaking, the samples were placed into an ultralow-temperature refrigerator (DW-86, Sysmedical, Shenyang, China) and frozen at −30 °C for 12 h. The samples were thawed at 25 °C for 12 h after freezing. The thawed samples were placed into a thermostatic reactor (BS, Labe Instrument, Shanghai, China) for alkali extraction at 90 °C for 2 h. Solid–liquid separation was carried out through a Brønsted funnel at the end of the reaction to obtain the extracted liquid and the remaining solid, respectively. The extracts were cooled and adjusted to pH 6 with hydrochloric acid and centrifuged at 3069× *g* in a centrifuge (ROTINA 420, Hettich, Tuttlingen, Germany) for 20 min, after which the supernatant was collected. The supernatant was added to three times the volume of anhydrous ethanol and left for 12 h. After centrifugation, the precipitate was washed two to three times with 75% ethanol and collected, and the precipitate was observed as being hemicellulose. Finally, hemicellulose was obtained via lyophilization by using a freeze dryer (Bench Top Pro, SP SCIENTIFIC, Warminster, PA, USA), after which it was ground and stored in a sealed container for use. The hemicelluloses that were obtained from bagasse, bamboo, corn straw, acacia, and eucalyptus raw materials were noted as being H-Bag, H-Bam, H-Corn, H-Aca, and H-Euc, respectively. The H composition analysis is shown in Table 1.

### 2.3. Enrichment of LCC

We weighed out 1 g absolute-dry samples of H-Bag, H-Bam, H-Corn, H-Aca, and H-Euc, respectively, and dissolved them in 70 mL, pH = 5, of citric acid–sodium citrate buffer solution to configure the hemicellulose solution. A total of 120 U·g^−1^ xylan endonuclease solution was added to the hemicellulose solution and enzymatically digested at 30 °C for 1 h [10]. The reaction was inactivated in a boiling water bath for 5 min and cooled to room temperature, and the supernatant was retained after centrifugation to remove the enzyme. XAD-16 N macroporous resin was added to the supernatant at a ratio of 1:30 lignin content to the absolute dry weight of the macroporous resin, for which we referred to a previous study for guidance on the procedure [7]. Afterwards, the samples were placed into an air shaker (ZQTY-50 N, SZ, Shanghai, China) and reacted at 30 °C and 150 rpm for 1 h. The macroporous resin and enzyme digest were obtained after filtration, separation, and adsorption. The enzymatic solution was stored at 4 °C. After adsorption, the surface of the macroporous resin was washed with distilled water and desorbed using three times the volume of methanol, after which the reaction was performed in an air shaker (30 °C, 150 rpm, 12 h). The macroporous resin was also desorbed by filtration and separated at the end of the reaction, and the desorbed solution was passed through a rotary evaporator (Interface l-100, BUCHI, Flawil, Switzerland) and freeze-dried to obtain a dried solid sample as LCC. The LCCs obtained from bagasse, bamboo, corn straw, acacia, and eucalyptus raw materials were denoted as LCC-Bag, LCC-Bam, LCC-Corn, LCC-Aca, and LCC-Euc, respectively.

### 2.4. Determination of Sugar Fraction

The sugar fraction was determined according to the National Renewable Energy Laboratory (NERL) standard method of detection [19], wherein solid samples were processed by using two-step acid hydrolysis. The following procedure was performed. First, the hemicellulose and LCC samples (50 mg) were accurately weighed and dissolved in 9 mL of 72% sulfuric acid. The samples were then shaken at 30 °C and 150 rpm for 1 h. After the first step, the concentration of sulfuric acid was diluted to 4% for the second step of acid hydrolysis, and the reaction was carried out at 121°C in a high-temperature autoclave for 1 h. The liquid after the two steps of acid hydrolysis was taken and centrifuged at a high speed for the test of sugar components. The sugar fractions in hemicellulose and LCC were determined by using ion chromatography (ICS-5000^+^, Thermo Fisher Scientific, Waltham, MA, USA) [20]. The mobile phase was 19% 200 mmol NaOH and 81% 18.25 MΩ ultrapure water. The detector was an ED electrochemical detector. The reference electrode was a gold electrode. The chromatographic column was a Dionex CarboPac PA20 column. The detector, reference electrode, and column were purchased from Thermo Fisher Scientific. The final data represent the average of the results of three identical experiments. The formula for calculating the content of the sugar fraction in the samples is shown in Equation (1):

(1) $$A=\frac{C\times 0.261\times n}{0.05}\times 100\%$$

where *A* is the relative % of monosaccharides in the sample; *C* is the monosaccharide concentration (g/L); *n* is the dilution number; 0.261 is the volume of solution (L) at acid digestion; and 0.05 is the absolute dry mass (g) of the acid digested sample.

The formula for calculating the xylose yield in the sample is shown in Equation (2):

(2) $$Y_{xyl}=(1-\frac{A_{1}\times m_{1}}{A_{2}\times m_{2}})\times 100\%$$

where *A*_1_ and *A*_2_ are the relative contents of xylose in the remaining solid and the raw material, respectively, and *m*_1_ and *m*_2_ are the absolute dry masses of the remaining solid and the raw material, respectively.

### 2.5. Lignin Content Determination

The solids (for acid-insoluble lignin content determination) and liquids (for acid-soluble lignin content determination) in the hydrolysate were separated after the two-step acid hydrolysis using a G4 glass filter. The final data were averaged from the results of three identical experiments.

For the determination of acid-soluble lignin content, the filtrate was diluted according to a certain ratio; moreover, according to the method of TAPPI [21], the absorbance value at 205 nm was measured by using a UV spectrophotometer (Cary 3500, Agilent, Santa Clara, CA, USA), and the absorbance range was fixed between 0.8 and 1.0 by controlling the dilution ratio. The formula for calculating the acid-soluble lignin content in the sample is shown in Equation (3):

(3) $$Acid-soluble\;lignin\left(\%\right)=\frac{n\times A\times 0.087}{110\times m_{0}}\times 100\%$$

where *n* indicates the dilution factor; *A* is the UV absorption value at 205 nm; 0.087 refers to the total volume of liquid during enzymatic digestion (L); 110 refers to the absorption coefficient; and *m*_0_ represents the absolute dry mass of the sample (g).

The acid-insoluble lignin (Klason lignin) content was determined as follows: the solid in the G4 filter was washed 3–4 times by using distilled water, the washed sample was dried at 105 °C to a constant weight, and the mass of the G4 filter was weighed before and after filtration.

The calculation formula is shown in Equation (4):

(4) $$Acid-insoluble\;lignin\left(\%\right)=\frac{m_{2}-m_{1}}{m_{0}}\times 100\%$$

where *m*_1_ represents the absolute dry mass of the filter before filtration (g); *m*_2_ indicates the absolute dry mass of the filter after filtration (g); and *m*_0_ is the absolute dry weight of the sample (g).

In addition, the total lignin content is calculated as shown in Equation (5):

(5) $$Total\;lignin\;content\left(\%\right)=Acid-soluble\;lignin\;\left(\%\right)+Acid-insoluble\;lignin\;\left(\%\right)$$

### 2.6. Molecular Weight Detection

A gel permeation chromatograph (GPC 50, Agilent, Santa Clara, CA, USA) was used to determine the heavy mean molecular weight (*M_w_*) and number mean molecular weight (*M_n_*) of LCC samples. The detector used in gel permeation chromatography is a differential refractive detector. As follows, 10 mg of LCC sample was dissolved in 10 mL of DMSO-*d*_6_ solution, shaken, and left to stand. One hundred microliters of the solution was injected into an Agilent PL-GPC220 for analysis. The final data are the average of the results of three identical experiments.

### 2.7. Nuclear Magnetic Resonance Spectroscopy Detection

The LCC sample was accurately weighed to 50 mg and dissolved in 0.7 mL of DMSO-*d*_6_ (99.8% D). The samples were examined by using a nuclear magnetic resonance spectrometer (AVANCE IIIHD500, Bruker, Saarbrücken, Germany) at 25 °C. NMR experiments were performed by using the Bruker ‘hsqcetgp’pulse program with 10,000 scans of ^13^C spectra and 100 scans for 2D HSQC-NMR experiments. The spectral widths were 12.9836 for 1SW and 19.9947 ppm for 2SW. Additionally, the content of the linkage bonds in LCC was calculated by using the combined ^13^C and HSQC NMR quantitative method, as proposed by Zhang et al. [22]. The calculation is shown in Equation (6):

(6) X100Ar=2Dx2DIS×CIS13C163-10313×600

where *X* is the semiquantitative structure; 2*D_x_* is the integral value of the 2D-HSQC signal of the structure to be quantified; *IS* is the overall integral region; ^13^*C*_163-103_ is the integral value of the aromatic ring region in the ^13^C spectrum; and 600 is the 600 aromatic carbons per 100 aromatic rings.

The semiquantitative ratios of the three lignin structural units in LCC were calculated as shown in Equation (7) [23]:

(7) $$G:S:H={2D}_{G2}:\frac{{2D}_{S2,6}}{2}:\frac{{2D}_{H2,6}}{2}$$

where 2*D_G_*_2_ is the 2D-HSQC integral value of the guaiacyl *G*_2_ signal; 2*D_S_*_2,6_ is the 2D-HSQC integral value of the lilacyl *S*_2,6_ signal; and 2*D_H_*_2,6_ is the 2D-HSQC integral value of the *p*-hydroxyphenyl *H*_2,6_ signal.

## 3. Results and Discussion

### 3.1. Analysis of LCC Components in Different Raw Materials

The composition of hemicellulose fractions of different raw materials is shown in Table 1. Comparing the yields of hemicellulose fractions from various plants, it was found that the present method extracted hemicellulose from plants with high extraction rates, ranging from 53.82% to 82.88%. Furthermore, the yield of hemicellulose in Gramineae was 69.56–82.88%, which was slightly higher than that in hardwood plants (53.82–64.79%). This indicated that hemicellulose was more easily extracted from Gramineae under the same treatment conditions. The results showed that the total sugar content of hemicellulose in gramineous plants was 50.02–64.65%, which was higher than that in hardwood plants (24.25–44.33%). A comparison of the monosaccharide contents of the fractions showed that the xylose content was the highest among all of the samples, thus indicating that xylose is the main chain sugar of hemicellulose in Gramineae and hardwoods. Moreover, the hemicellulose arabinose content in Gramineae (4.83–11.18%) was significantly higher than that in hardwood hemicelluloses (0.74–0.94%). The results showed that the hemicellulose in gramineous plants had a higher degree of branched chains and more branched structures in the main chain than in hardwood. In addition, the content of glucose in bamboo is much higher than that in other raw hemicelluloses. The results of Felisberto et al. [24] suggested that a large amount of starch is present in bamboo and that this fraction of starch is readily soluble in water, which leads to a higher glucose content in bamboo hemicellulose than in various other plants. The results of the analysis of lignin in hemicellulose demonstrated that the total lignin content of eucalyptus hemicellulose was 21.40%, which was much higher than the total lignin content in the remaining four hemicellulose samples (9.97–11.65%). Additionally, the acid-soluble lignin content in eucalyptus hemicellulose reached 12.93% compared to the remainder of the hemicelluloses, which was much higher than the content of the remaining hemicelluloses (3.01–4.60%). A large amount of lignin was present in the alkaline extract, and during pH adjustment and acid precipitation, a large amount of lignin precipitated down. Thus, the extraction yields hemicellulose with a low klason lignin content. This was the main factor contributing to the high lignin content in eucalyptus hemicellulose.

The composition of LCC fractions of different raw materials is shown in Table 1. LCC mainly consisted of xylose, arabinose, glucose, and lignin. In the analysis of hemicellulose, we can find that the sugar fraction in hemicellulose is mainly dominated by xylose. The sugar content of LCC extracted by xylanase and macroporous resin was also dominated by xylose. The sugar content of LCC extracted by xylanase and macroporous resin was significantly reduced, and the lignin content of LCC was also significantly increased. Analysis of the sugar fractions in LCC demonstrated that the sugar content of LCC from Gramineae (47.06–55.01%) was significantly higher than that of LCC from hardwoods (20.83–34.15%); additionally, the xylose content was the highest among the sugar fractions of LCC samples from all of the raw materials. In addition, the LCC of Gramineae contained more arabinose than that of hardwoods. Related studies have shown that arabinose in Gramineae is linked to lignin via ferulic acid esters [8]. Therefore, the conclusion that there is a significant amount of arabinose in LCC was consistent with the experimental results of previous studies. Glucose content in the LCC of grasses (4.30–10.44%) was also higher than in the LCC of hardwoods (1.80–1.86%). In contrast, galactose content was lower in the five LCC species, ranging from 0.65% to 2.25%. Side chain sugar content was significantly higher in graminoids than in hardwoods, indicating a high degree of LCC branching in graminoids. Moreover, the analysis of the lignin fraction of LCC in different raw materials suggested that the lignin fraction mainly consisted of acid-soluble lignin and acid-insoluble lignin. The lignin content of hardwood LCC (19.5–29.28%) was higher than that of Gramineae LCC (8.94–16.31%), and the findings were consistent with the lignin content of hemicellulose, thus indicating that the lignin fraction of hemicellulose was the main source of lignin in LCC. In addition, the lignin content of eucalyptus LCC was higher than the sugar fraction content, whereas the sugar fraction content was higher than the lignin content in the remaining species of LCC; these findings were related to the composition of hemicellulose components.

The experimental results demonstrated that the LCC extracted from different raw materials had a large variability in composition. Ara:Glu ≈ 1:3 in all Gramineae LCC, thus indicating that after enzymatic digestion by xylan endonuclease, hemicellulose carried three xylose on the arabinose attached to lignin in the LCC obtained by adsorption on macroporous resin. Furthermore, it was found that the various monosaccharide fractions were more abundant in the LCC of Gramineae than in hardwood LCC, thus suggesting that the LCC of Gramineae may contain diverse LC bonds.

**Figure 2:**
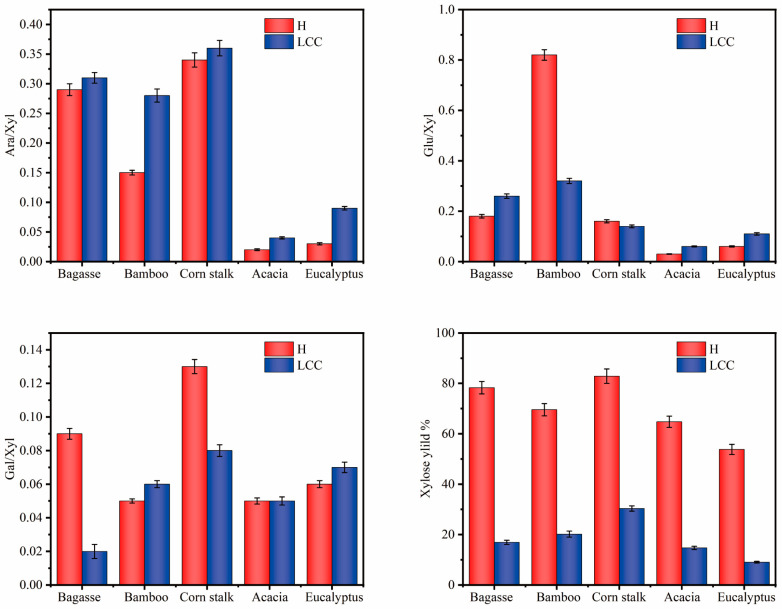
Ratio of each sugar component to xylose and yield of xylose.

By comparing the molar ratio of the monosaccharide content to the xylose content in hemicellulose and LCC in Figure 2, it is possible to analyze the changes in the proportionality of each type of sugar to xylose in hemicellulose and LCC. Among them, the ratio of arabinose to xylose represents the degree of branching of hemicellulose; specifically, a higher degree of branching corresponds to a higher solubility of polysaccharides. When comparing the Ara/Xly values of hemicellulose and LCC, it can be observed that the Ara/Xly values in LCC are generally higher than those in hemicellulose. The ratio of arabinose to xylose in Gramineae hemicellulose (0.15–0.29) was much higher than that of arabinose to xylose for hardwood hemicellulose (0.03–0.06). This indicated that Gramineae hemicellulose was more soluble than hardwood hemicellulose [25]. When comparing the values of Gal/Xyl in hemicellulose and LCC, it was observed that the values of Gal/Xyl in LCC of all plants were lower than those in hemicellulose, except for the increased values of Gal/Xyl in bamboo LCC. Moreover, when comparing the Glu/Xyl values of hemicellulose and LCC, it was found that the values of Glu/Xyl in maize straw LCC and bamboo LCC were lower than those in hemicellulose. This effect was attributed to the high glucose content in hemicellulose due to the high starch content in bamboo [24]. The raw material was freeze–thawed and then extracted to obtain hemicellulose after treatment with sodium hydroxide for 2 h at 90 °C. The yield of hemicellulose was in the range of 53.82–82.88%.

Furthermore, the main chain of hemicellulose primarily consists of xylan. During the extraction process, the main chain long-chain hemicellulose was cut off by xylanase to become a short-chain structure, and the short-chain structure with lignin was subsequently adsorbed out by using a macroporous resin, thus resulting in a lower xylose content. The extraction rate of xylose in the five LCCs ranged from 9.02% to 30.34%, which demonstrated the effective treatment of hemicellulose by xylanase. The experimental results demonstrated that a large amount of hemicellulose could be extracted by freeze-thaw-assisted alkali treatment, thus making the extracted LCCs more strongly representative.

### 3.2. Analysis of the Molecular Weight of LCC in Gramineae

Table 2 shows that the M_w_ of the five LCCs ranged from 1567 g·mol^−1^ to 3587 g·mol^−1^, M_n_ ranged from 970 g·mol^−1^ to 2234 g·mol^−1^, and the molecular weights of the Gramineae LCCs were significantly higher than those of the hardwood LCCs. This effect was attributed to the relatively low percentage of sugar content in LCC-Aca and LCC-Euc, as well as the larger molecular weight of the sugar fraction in hemicellulose compared to alkali lignin, thus resulting in a relatively small molecular weight of hardwood LCC [26]. Xie [18] and Su [27] examined the M_w_ and M_n_ of LCC by using different methods, and their reported M_w_ was approximately 7907–20,748 and the M_n_ was approximately 4105–12,802, much higher than the M_w_ and M_n_ of LCC obtained in this study, thus indicating that the different extraction methods of LCC were directly responsible for their molecular weight sizes. The relative molecular weights of LCCs that have been obtained from different plant hemicelluloses treated with xylan endonuclease were generally small. Moreover, the polydispersity M_w_/M_n_ of the five plant LCCs ranged from 1.426 to 1.615, thus exhibiting a relatively narrow molecular weight distribution with a polydispersity index (M_w_/M_n_) less than 2.0. A more narrow polydispersity indicates better physicochemical stability, which is one of the important properties of lignin polymers [28]. This effect also demonstrated the better solubility of the five LCCs in the NMR solvent (DMSO-d_6_), which facilitated the detection of the NMR signal of the LC bond [29]. The experimental results demonstrated that the carbohydrate content in the LCC of Gramineae was higher than that in the LCC of hardwood plants, which corroborated the results of the analysis of Gramineae and hardwood plant fractions.

### 3.3. Two-Dimensional NMR Spectroscopy of LCC Enrichment in Gramineae

Nuclear magnetic resonance spectroscopy is a widely used tool for the analysis of polysaccharide structures [30] and offers significant advantages in investigating analyses of LCC structures. The application of 2D NMR techniques circumvents the problem of signal peak overlap, and the accurate quantification of the LCC structure via 2D and ^13^C NMR spectroscopy is a promising approach to analyze the LCC structure [8]. The NMR signals of the carbohydrate part, lignin part, and PhGlc bond part of the LCC structure were mainly observed in the vicinity of the side chain region (δ_C_/δ_H_ 95~50/5.0~2.5 ppm), benzene ring region (δ_C_/δ_H_ 145~100/7.8~6.0 ppm), and PhGlc bond (δ_C_/δ_H_ 105~96/5.2~4.0 ppm) in the NMR pattern region. The NMR signals and chemical shifts of all the structures were determined according to the methodology of previous studies, and the signal assignments are shown in the Supporting Materials (S1).

#### 3.3.1. NMR Analysis of Carbohydrate Fractions

**Figure 3:**
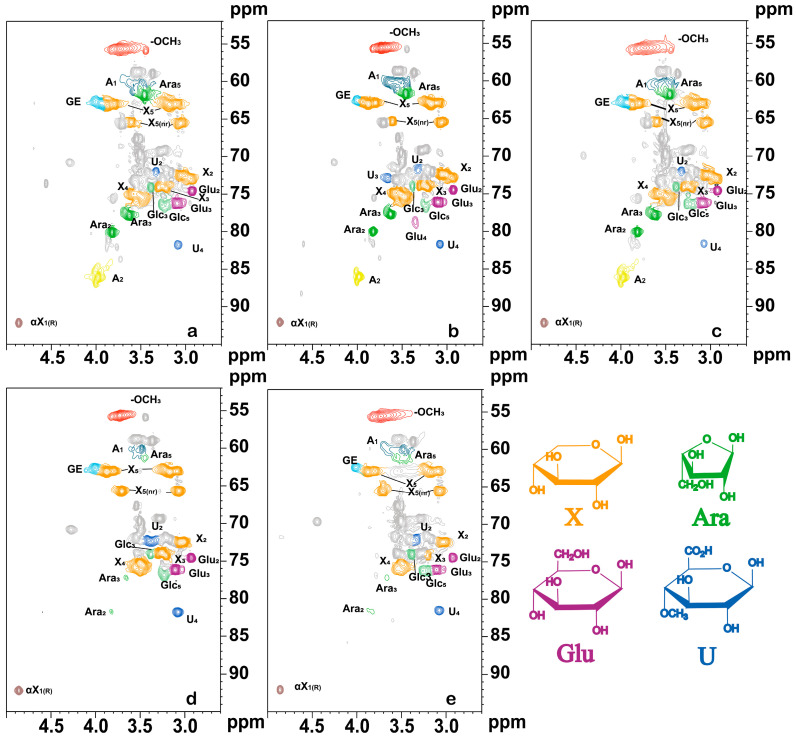
The 2D-NMR side-chain spectra under different plant LCC conditions. (**a**). LCC-Bag; (**b**). LCC-Bam; (**c**). LCC-Corn; (**d**). LCC-Aca; (**e**). LCC-Euc.

As shown in Figure 3, abundant carbohydrate-related NMR signals can be observed in the side chain region of the 2D HSQC spectra for different feedstock LCCs. For all the LCC samples, correlated signals of β-D-xylan C_2_-H_2_, C_3_-H_3_, C_4_-H_4_, and C_5_-H_5_ were observed at δ_C_/δ_H_ 82.5/3.02, 73.7/3.22, 75.4/3.60, 6.26/3.40, and 3.72 ppm, respectively, and the signal of C_5_-H_5_ at the nonreducing end of β-D-xylan could be observed at δ_C_/δ_H_ 65.3/3.61 and 3.05 ppm. When combining the results of the analysis of all of the LCC fractions, it was evident that the relative content of xylose was the highest in all of the LCC samples; therefore, the signal peaks of xylose in the side chain region were more significant than those of other monosaccharides. In addition, the signal peaks of xylose were more prominent in the LCC of Gramineae compared to the LCC carbohydrate signal peaks in hardwoods. Moreover, the signal peaks C_2_-H_2_, C_3_-H_3_, and C_5_-H_5_ (δ_C_/δ_H_ 80.0/3.83, 77.7/3.63, and 61.6/3.46 ppm, respectively) of α-L-furan-type arabinose in LCC of Gramineae were observed at a very significant rate; however, the signal peaks C_2_-H_2_ and C_3_-H_3_ of α-L-furan-type arabinose in hardwood LCC disappeared, and the α-L-furan-type arabinose signal peak C_5_-H_5_ had very weak signal intensity. In combination with the results of LCC composition analysis, only a small amount of arabinose was present in the hardwood LCC, which may be related to the composition of hemicellulose in the raw material. Among them, Gramineae hemicellulose was composed of arabinoxylan-4-O-methylglucuronide xylose, whereas hardwood hemicellulose was composed of poly-O-acetyl-4-O-methylglucuronide xylose. Additionally, xylanase treatment of hemicellulose preserved arabinose in the hemicellulose of Gramineae plants; thus, considerable amounts of arabinose were observed in both fraction analysis and NMR. The signal peaks of C_2_-H_2_ and C_3_-H_3_ in β-D-glucopyranose-type glucose in five plant LCCs were observed at δ_C_/δ_H_ 74.4/2.93 and 76.2/3.09, respectively. The C_2_-H_2_ and C_4_-H_4_ signals of 4-O-methyl-α-D-glucuronic acid were observed at δ_C_/δ_H_ 71.9/3.33 and 81.6/3.08 ppm, respectively. Signal peaks of C_4_-H_4_ (δ_C_/δ_H_ 78.6/3.36) in β-D-glucopyranose-type glucose and C_3_-H_3_ (δ_C_/δ_H_ 72.9/3.66) in 4-O-methyl-α-D-glucuronic acid were clearly observed in bamboo LCC due to the presence of a large amount of glucose in the composition of bamboo LCC, which is more structurally rich. In the 2D HSQC NMR characterization, it can be found that the signal peaks of carbohydrates in the LCC of graminoid plants are higher than those of hardwood plants, indicating a high percentage of carbohydrates in the LCC of graminoid plants.

#### 3.3.2. NMR Analysis of the Lignin Fraction

**Figure 4:**
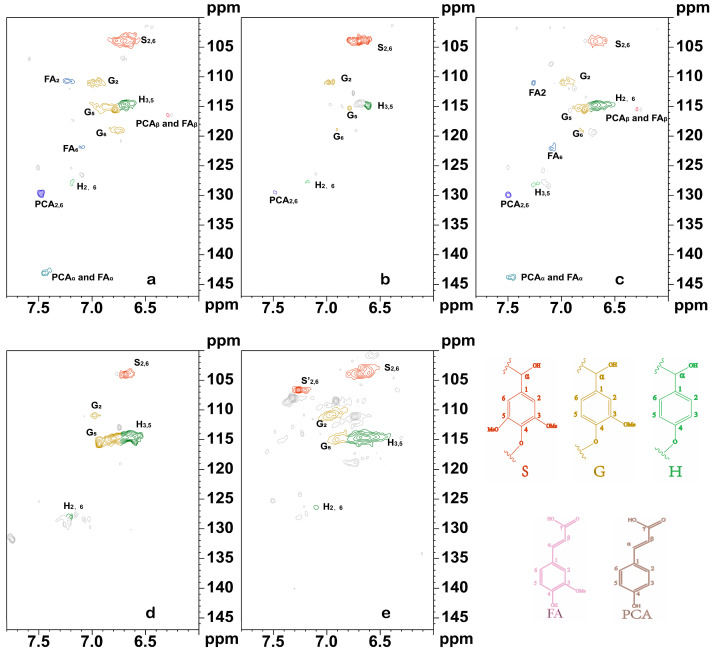
The 2D-NMR lignin fractions under different plant LCC conditions. (**a**). LCC-Bag; (**b**). LCC-Bam; (**c**). LCC-Corn; (**d**). LCC-Aca; (**e**). LCC-Euc; S: syringyl units; G: guaiacyl units; H: *p*-hydroxyphenyl units; FA: ferulate substructures; PCA: *p*-coumarate substructures.

As shown in Figure 4, the relevant NMR signals of the lignin fraction in LCC were mainly found in the benzene ring region. The signal peaks of three lignin structural units (syringyl S, guaiacyl G, and *p*-hydroxyphenyl H) in the benzene ring region were observed in all of the LCC samples. Among them, the C_2,6_-H_2,6_ cross-signal peak attributed to the lilac-based unit was observed at δ_C_/δ_H_ 103.97/6.69 ppm. Moreover, the C_2_-H_2_ cross-signal peak attributed to the G-unit was observed at δ_C_/δ_H_ 110.8/6.96 ppm, and the C_2,6_-H_2,6_ cross-signal peak attributed to the p-hydroxyphenyl unit at δ_C_/δ_H_ 127.79/7.22 ppm was also observed. The quantitative method combining quantitative ^13^C NMR and 2D NMR, as proposed by Wen et al. [23], was used to calculate the semiquantitative integration of the 2D NMR cross-signal peaks of the three lignin structural units, and the results are shown in Table 3. Among the types and contents of lignin in various plant LCCs, the lignin structural units of LCC-Bag, LCC-Bam, and LCC-Aca exhibited a pattern of S > G > H. The content of lignin structural units in LCC-Corn and LCC-Euc exhibited a pattern of G > S > H, wherein the G-unit and S-unit lignin contents were close to each other. Furthermore, H-unit lignin structural units were less abundant in various plant LCCs, which may be related to the fact that H-unit lignin structural units are attached to lignin macromolecules by ester bonds [31]. As in the alcohol precipitation process, the low-molecular-weight LCC (mainly S-units) was precipitated together with most carbohydrates [26]. Moreover, the ester bond is unstable in alkaline environments, thus resulting in a large amount of destruction of H-unit structural units connected by ester bonds and making them less abundant in LCC samples [8]. The experimental data demonstrated that the lignin in the LCC of all five plant species was dominated by S-unit and G-unit, and the H-unit lignin content was scarce. The feedstock differences led to some variability in the lignin content of the LCC extracted under an alkaline environment. For example, the S-unit content in LCC-Bam was twice as high as the G-unit content. The remaining four LCC samples showed little difference between S-unit content and G-unit content; additionally, the H-unit content in hardwood LCC was lower than that in Gramineae LCC. It has been reported that the main connections between lignin parts in LCC were β-O-4, β-β, β-5, and β-1 bonds [28]. Moreover, the linkages between lignin in various plant LCCs obtained after xylanase enzymatic digestion were observed and found to mainly include β-O-4 structures, with a few β-β and β-5 linkages observed in hardwood plant LCCs and no β-1 linkages being found. In addition, the most common interunit connection of β-O-4 was connected by the S-unit of lignin. The analysis of the interunit connections between lignin demonstrated that the β-O-4 structure was the main connection between lignin in the extracted LCC. The β-O-4 structure was more abundant in hardwoods (48.07–55.19 per 100 Ar) than in Gramineae plants (22.13–45.57 per 100 Ar). As shown in Figure 2, A_2_ can be observed in Gramineae LCC at 86.3/4.02 ppm, which is the signal peak of C_β_-H_β_ of the β-O-4 structure linked to S-unit [20,32]. This scenario may be due to the higher purity of the extracted hardwood LCC than the Gramineae LCC. The signal peak of A_1_ can be observed at δ_C_/δ_H_ 55.8–62.1/3.40–3.70 ppm, which is attributed to the C_γ_-H_γ_ of the β-O-4 structure. The signal peak of A_2_ can be observed at δ_C_/δ_H_ 86.3/4.02 ppm, which is generated by the Cβ-Hβ of the β-O-4 structure linked to the S-type lignin. Furthermore, A_1_ and A_2_ are present in the Gramineae, and no signal peak of A_2_ was found in the hardwood LCC. The analysis demonstrated that C_β_ and C_γ_ would be connected through the β-O-4 structure in Gramineae LCC. In hardwood plant LCC, only C_γ_ was connected by the β-O-4 structure. The β-β and β-5 connections were almost absent in LCC-Bag and LCC-Bam. However, few β-β and β-5 structures are present in LCC-Corn and LCC-Euc. In summary, except for the corn straw LCC, there are fewer lignin bonds and fewer linkages between lignin in the Gramineae LCC than in the hardwood LCC. In alkaline environments, the connections between plant LCC lignin are mainly in β-O-4 linkages. In alkaline environments, lignin in the LCC of graminoids and the LCC of hardwoods is mainly dominated by S-unit lignin and G-unit lignin, which are connected to each other by means of β-O-4.

The signals of ferulic acid ester units C_2_-H_2_ (δ_C_/δ_H_ 111.06/7.30 ppm) and C_6_-H_6_ (δ_C_/δ_H_ 120.61/7.04 ppm) were detected in LCC-Bag and LCC-Corn. The signal for the coumarin ester unit C_2,6_-H_2,6_ (δ_C_/δ_H_ 129.73/7.48 ppm) was detected in LCC-Bag, LCC-Bam, and LCC-Corn. Moreover, the signals of C_α_-H_α_ (δ_C_/δ_H_ 143.85/7.47 ppm) and C_β_-H_β_ (δC/δH 115.17/6.27 ppm) for coumarin and ferulic acid ester units were observed in LCC-Bag and LCC-Corn. The structure of the p-coumarin ester unit C_2,6_-H_2,6_ and the signal peaks of the *p*-coumarin ester and ferulic acid ester unit C_α_-C_β_ can be clearly observed in LCC-Bag, LCC-Bam, and LCC-Corn. In contrast, hardwoods do not have such a structure as that described above, thus demonstrating that lignin–ferulic acid ester/p-coumaric acid ester-arabinose-based xylan is a type of linkage in non-wood plant LCC under alkaline conditions, and that this structure does not exist in hardwood LCC. Related LCC studies have shown that ferulate is a cross-linker between arabinoxylan and lignin [33].

#### 3.3.3. NMR Analysis of LC Bonding Components

PhGlc bonds, BE bonds, and benzyl ester bonds are the three most common natural LC bonds in plants [34]. As shown in Table 4, the highest content of PhGlc bonds was found in LCC-Bam, followed by LCC-Aca, LCC-Bag, and LCC-Euc; additionally, the lowest amount of PhGlc bonds was found in LCC-Corn. A small amount of BE bonds exists in LCC-Bag and LCC-Euc. The five LCC PhGlc bonds’ contents ranged from 10.75 to 25.06 (per 100 Ar). BE bond content was detected in LCC-Bag and LCC-Euc at 0.42 (per 100 Ar) and 0.14 (per 100 Ar), respectively. The experimental results demonstrated that the PhGlc bond was a type of LC bond that could exist stably in both strong and alkaline environments. The PhGlc bond was the main connecting bond of the LCC structure under alkaline conditions for different raw materials of LCC.

**Figure 5:**
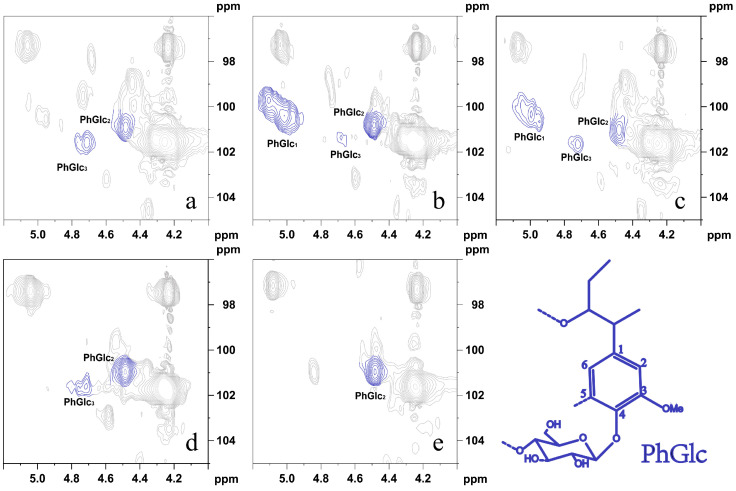
The 2D-NMR phenyl glycoside bonds under different plant LCC conditions. (**a**). LCC-Bag; (**b**). LCC-Bam; (**c**). LCC-Corn; (**d**). LCC-Aca; (**e**). LCC-Euc.

It was demonstrated that the type of lignin structural unit in the PhGlc bond and the lignin-linked sugar fraction affect their signal peaks in 2D NMR [35]. As shown in Figure 5, the cross-signal peaks of three types of PhGlc bonds (PhGlc_1_, PhGlc_2_, and PhGlc_3_) could be observed at δ_C_/δ_H_ 100.06/5.03 ppm, δ_C_/δ_H_ 101.01/4.46 ppm, and δ_C_/δ_H_ 99.53/4.75 ppm, respectively.

This indicated that different sugar components form three types of glycosidic-type linkage bonds with phenolic hydroxyl groups in lignin. Cross-signal peaks of three types of PhGlc bonds (PhGlc_1_, PhGlc_2_, and PhGlc_3_) were present in LCC-Bam and LCC-Corn, whereas only one type of PhGlc bond (PhGlc_2_) was present in hardwood LCC. Cross-signal peaks of two types of PhGlc bonds (PhGlc_2_ and PhGlc_3_) were present in LCC-Bag and LCC-Aca. As shown in Table 5, PhGlc_1_ in LCC-Bam and LCC-Corn had a high proportion in the total PhGlc bond class. The PhGlc bond contents in LCC-Bam and LCC-Corn were ranked as PhGlc_2_ > PhGlc_1_ > PhGlc_3_ and PhGlc_2_ > PhGlc_3_ in LCC-Bag and LCC-Corn. Moreover, only PhGlc_2_ was present in LCC-Euc. Miyagawa et al. [36] simulated the basic NMR data of PhGlc carbohydrate complexes by synthesizing 12 monoglycol β-glycosides. It was hypothesized that the main sugar attached to PhGlc_1_ in the extracted LCC was glucose, with the main sugars attached to PhGlc_2_ being xylose, glucose, and galactose, and the main sugar attached to PhGlc_3_ being mannose. Combined with the results of the LCC composition analysis, it was found that the hardwood LCC contained more xylose in the sugar composition and very few other sugars; therefore, it is presumed that the sugar composition of PhGlc_2_ is related to xylose. In addition, the highest xylose content was found in the LCC of Gramineae, and the highest PhGlc_2_ content was likewise detected in the NMR results. The experimental results showed that there are more types of PhGlc bonds in graminaceous LCC than in hardwood plant LCC, and that the sugar composition of PhGlc_2_ is related to xylose.

**Figure 6:**
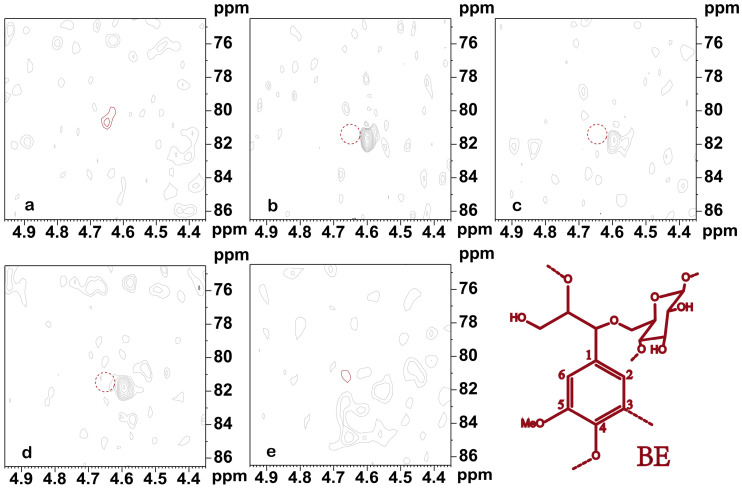
The 2D-NMR BE components under different plant LCC conditions. (**a**). LCC-Bag; (**b**). LCC-Bam; (**c**). LCC-Corn; (**d**). LCC-Aca; (**e**). LCC-Euc.

As shown in Figure 6, the BE bond signal peak was identified at δ_C_/δ_H_ 81.3/4.65 ppm, based on relevant studies. However, only a small amount of BE bonds (BE) can be observed in LCC in alkaline environments. No BE bonds were detected in LCC-Bam, LCC-Corn, and LCC-Aca. This is attributed to both the LC ether and LC ester bonds all being alkali-sensitive linkages and alkali treatment being able to break the ether and ester linkages between lignin, hemicellulose, and cellulose as compared to acid or oxidative processes. A few LC ether bond signal peaks could be observed in plant LCC under five alkaline conditions; however, no LC ester bond signal peaks were found because the LC ether bond is more stable than the LC ester bond [37]. The experimental results demonstrated that the LCC in plants was enriched by macroporous resin in an alkaline environment, wherein the LC bonds were generally PhGlc bonds, and BE and benzyl ester bonds were almost absent. The LCC side-chain sugar fractions of Gramineae are high in content, with more PhGlc bond types.

## 4. Conclusions

In this study, the LCC structures of hardwoods and grasses were enriched under alkaline conditions, and the similarities and differences in LCC structures between different raw materials after alkaline treatment were discussed and analyzed. The experimental results suggested that the monosaccharide fraction of LCC was more abundant in Gramineae than in hardwoods. Moreover, the G-unit and S-unit were mainly dominant in the lignin of LCC of Gramineae, and the lignin was mainly connected by β-O-4. The number of PhGlc bonds in the LCC of grasses and hardwood plants ranges from 10.75 to 25.06 (per 100 Ar). The LC bonds of LCC species in Graminaceous plants were mainly dominated by three phenyl glycosidic bonds, and the sugar composition of PhGlc_1_ and PhGlc_3_ was related to side-chain sugars. Furthermore, the phenyl glycosidic bonds in hardwood LCC were mainly dominated by PhGlc_2_, and the sugar composition of PhGlc_2_ was related to xylose. The results show the similarities and differences in the structure of hardwood and grass LCCs in alkaline environments and provide theoretical data for realizing the high-value utilization of woody resources.

## Figures and Tables

**Table 1:** Analysis of different plant hemicelluloses and LCC fractions.

Sample	TS (%)	TL (%)	Ara (%)	Gal (%)	Glu (%)	Xyl (%)	Asl (%)	Kl (%)
H-Bag	59.36	11.44	11.18	3.35	6.77	38.06	3.11	8.33
H-Bam	64.65	9.97	4.83	1.53	26.29	31.99	3.01	6.96
H-Corn	50.02	11.65	10.41	4.12	4.80	30.68	4.60	7.05
H-Aca	44.33	11.00	0.94	1.95	1.14	40.30	3.37	7.63
H-Euc	24.25	21.40	0.74	1.18	1.17	21.16	12.93	8.47
LCC-Bag	55.01	8.94	10.81	0.65	9.03	34.52	4.98	3.96
LCC-Bam	53.77	12.16	9.05	2.09	10.44	32.18	5.20	6.96
LCC-Corn	47.06	16.31	10.79	2.25	4.30	29.71	6.68	9.64
LCC-Aca	34.15	19.50	1.24	1.49	1.86	29.57	5.75	13.75
LCC-Euc	20.83	29.28	1.54	1.19	1.80	16.30	16.26	13.02

TS = total sugar, TL = total lignin, Ara = Arabinose, Gal = galactose, Glu = Glucose, Xyl = Xylose, Asl = Acid soluble lignin, Kl = Klason lignin.

**Table 2:** Differences in molecular weight of LCC in different plants.

Sample	M_w_ (g·mol^−1^)	M_n_ (g·mol^−1^)	M_w_/M_n_
LCC-Bag	3587	2234	1.605
LCC-Bam	3229	2062	1.566
LCC-Corn	2826	1905	1.483
LCC-Aca	2198	1541	1.426
LCC-Euc	1567	970	1.615

**Table 3:** Lignin species and linkage patterns in LCC of different plants.

Features	LCC-Bag	LCC-Bam	LCC-Corn	LCC-Aca	LCC-Euc
G:S:H	6.4:8.2:0.7	2.3:5.3:1.5	5.2:4.2:1.2	2.4:2.8:0.5	7.9:7.1:0.4
β-O-4	40.63	45.57	22.13	55.19	48.07
β-β	ND	ND	5.44	5.28	6.50
β-5	ND	ND	0.73	ND	2.96

**Table 4:** Analysis of LC bonds in LCC of different plants.

Signal	LCC-Bag	LCC-Bam	LCC-Corn	LCC-Aca	LCC-Euc
PhGlc	14.24	25.06	10.75	16.89	12.56
BE	0.42	ND	ND	ND	0.14

**Table 5:** Semiquantitative analysis of three PhGlc bonds in LCC of different plants.

Signal	LCC-Bag	LCC-Bam	LCC-Corn	LCC-Aca	LCC-Euc
PhGlc_1_	ND	9.26	3.53	ND	ND
PhGlc_2_	11.65	15.51	6.38	14.82	12.56
PhGlc_3_	2.59	0.29	0.84	2.07	ND

## Data Availability

Data are contained within the article.

## Supplementary Materials

The following supporting information can be downloaded at: https://www.mdpi.com/article/10.3390/polym16101403/s1, Supporting Materials S1: 2D-NMR HSQC signal peak attribution.
