# Supplementary material for: Differential Studies on the Structure of Lignin–Carbohydrate Complexes (LCC) in Alkali-Extracted Plant Hemicelluloses
Source: Polymers (Basel). 2024 May 15;16(10):1403. doi: 10.3390/polym16101403 (PMC11124851; doi:10.3390/polym16101403)
Supplement: Supplementary file 1 [file polymers-16-01403-s001.zip › polymers-3015809-supplementary.pdf]

S 1 2D-NMR HSQC signal peak attribution

Label	δ_C/δ_H (ppm)	Assignment
$-OCH_3$	55.6/3.73	C-H in methoxyls
Ara_2	80.0/3.83	C_2-H_2 in α-L-Arabinofuranoside
Ara_3	77.7/3.63	C_3-H_3 in α-L-Arabinofuranoside
Ara_5	61.6/3.46	C_5-H_5 in α-L-Arabinofuranoside
X_2	72.5/3.02	C_2-H_2 in β-D-xylopyranoside (X)
X_3	73.7/3.22	C_3-H_3 in β-D-xylopyranoside (X)
X_4	101.5/4.26	C_4-H_4 in β-D-xylopyranoside (X)
X_5	62.6/3.72 and 3.40	C_5-H_5 in β-D-xylopyranoside (X)
$X_{5(nr)}$	65.3/3.61 and 3.05	C_5-H_5 in β-D-xylopyranoside of xylans non reducing end
Glu_2	74.4/2.93	C_2-H_2 in β-D-glucopyranoside
Glu_3	76.2/3.09	C_3-H_3 in β-D-glucopyranoside
Glu_4	78.6/3.36	C_4-H_4 in β-D-glucopyranoside
Glc_3	73.9/3.38	C_3-H_3 correlations from anomeric positions of glucans (G)
Glc_5	76.3/3.20	C_5-H_5 correlations from anomeric positions of glucans (G)
U_2	71.9/3.33	C_2-H_2 in 4-O-methyl-α-D-GlcUA
U_3	72.9/3.66	C_3-H_3 in 4-O-methyl-α-D-GlcUA
U_4	81.6/3.08	C_4-H_4 in 4-O-methyl-α-D-GlcUA
A_1	62.1~55.8/3.70~3.40	C_γ-H_γ in β-O-4 substructures (A)
A_2	86.3/4.02	C_β-H_β in β-O-4 substructures linked to S units (A)
GE	62.7/4.01	γ-ester linkages
$PhGlc_1$	100.4/5.01	phenyl glycoside linkages
$PhGlc_2$	100.5/4.46	phenyl glycoside linkages
$PhGlc_3$	99.53/4.75	phenyl glycoside linkages
$S_{2,6}$	103.9/6.69	$C_{2,6}$-$H_{2,6}$ in etherified syringyl units (S)
$S'_{2,6}$	105.8/6.95	$C_{2,6}$-$H_{2,6}$ in oxidized (αC=O) syringyl units (S)
G_2	110.8/6.96	C_2-H_2 in guaiacyl units (G)
G_5	115.6/6.92	C_2-H_2 in guaiacyl units (G)
G_6	118.1/6.96	C_6-H_6 in guaiacyl units (G)
$H_{2,6}$	127.8/7.22	$C_{2,6}$-$H_{2,6}$ in p-hydroxyphenyl units
$H_{3,5}$	114.6/6.62	$C_{3,5}$-$H_{3,5}$ in p-hydroxyphenyl units
FA_2	111.1/7.31	C_2-H_2 in ferulate (FA)
FA_6	120.6/7.04	C_6-H_6 in ferulate (FA)
$PCA_{2,6}$	129.7/7.48	C_2-H_2 and C_6–H_6 inp-coumarate (PCA)

PCA_{α}/FA_{α}	143.9/7.47	C_{α}-H_{α} in p-coumarate (PCA) and ferulate (FA)
PCA_{β}/FA_{β}	115.2/6.27	C_{β}-H_{β} in p-coumarate (PCA) and ferulate (FA)
